# Co‐Infection, but Not Infection Intensity, Increases Shedding in a Gastrointestinal Helminth of Gamebirds

**DOI:** 10.1002/ece3.71483

**Published:** 2025-06-05

**Authors:** Katherine Prescott, Emile Michels, Barbara Tschirren

**Affiliations:** ^1^ Centre for Ecology and Conservation University of Exeter Penryn UK

**Keywords:** disease ecology, host–parasite interactions, infectious disease transmission, supershedders, virulence evolution, wildlife disease dynamics

## Abstract

Host heterogeneity in disease transmission is commonly seen across host‐pathogen systems, and identifying individuals who contribute disproportionately to pathogen transmission (i.e., superspreaders) is key to understanding disease dynamics and managing outbreaks. It is often assumed that shedding intensity is directly proportional to infection intensity. However, theory predicts that co‐infection might modulate the relationship between infection intensity and shedding, promoting increased onward transmission. Here, we quantify the relative importance of infection intensity and co‐infection on shedding in *Heterakis gallinarum*, a gastrointestinal helminth of gamebirds, in a population of ring‐necked pheasants during the shooting season of 2023. We found that infection intensity was a poor predictor of shedding intensity. Instead, increased shedding was linked to co‐infections with other endoparasites. Hosts co‐infected with *Syngamus trachea, Capillaria* spp. and/or *Eimeria* spp. exhibited higher shedding intensity of *H. gallinarum* than those infected with *H. gallinarum* alone. This effect was additive, with each additional co‐infection further increasing *H. gallinarum* shedding. There were no sex differences in shedding, but male hosts had higher *H. gallinarum* infection intensities. Our results show that shedding intensity is not simply explained by infection intensity, but rather is the result of complex host–parasite and parasite–parasite interactions. This highlights the importance of considering such interactions in understanding disease emergence and persistence in natural populations.

## Graphical abstract

Host heterogeneity in disease transmission is influential in shaping disease dynamics, but its drivers remain poorly understood. Shedding intensity is often assumed to be directly proportional to infection intensity; however, we found that infection intensity was a poor predictor of shedding intensity in Heterakis gallinarum, a gastrointestinal helminth of gamebirds. Instead, increased shedding was linked to co‐infections with other endoparasites, highlighting the importance of complex host–parasite and parasite–parasite interactions for disease transmission dynamics.

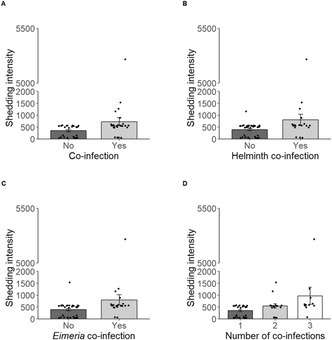

## 1 Introduction

Across host–parasite systems, hosts display marked inter‐individual heterogeneity in their contribution to onward transmission of disease (Lloyd‐Smith et al. 2005; Lloyd et al. 2020; VanderWaal and Ezenwa 2016; Woolhouse et al. 1997). Transmission heterogeneity is generalised into the 80:20 rule, where 80% of infections stem from 20% of hosts (Lloyd‐Smith et al. 2005; Stein 2011; Sun et al. 2021). This minority of hosts responsible for the majority of infections are referred to as ‘superspreaders’ (see Table 1 for definition) (Lloyd‐Smith et al. 2005; Matthews et al. 2009; Woolhouse et al. 1997). Superspreaders are critical in shaping the dynamics of disease outbreaks (Garske and Rhodes 2008; Lloyd‐Smith et al. 2005; Mohindra et al. 2021).

**TABLE 1:** Definitions of key terms used in this study.

Term	Definition
Infectiousness	The capacity of an infected host to infect naïve individuals (Cattadori et al. 2014, Graham et al. 2007)
Shedding intensity	The degree to which a host secretes infectious agents into the external environment. Encapsulates one aspect of infectiousness.
Supershedders	An individual that sheds a disproportionately high level of infectious agents into the environment (Lass et al. 2013).
Superspreaders	An individual that contributes more to onward transmission than the 99^th^ percentile of infected hosts (Lloyd‐Smith et al. 2005).

The drivers of transmission heterogeneity are still poorly understood, but variation in host infectiousness, the capacity of a host to infect naive individuals (see Table 1 for definition), likely plays a key role (Cattadori et al. 2014; Graham et al. 2007). Shedding is a key component of infectiousness: highly infectious hosts exhibit higher shedding intensities (see Table 1 for definition) of transmissible elements into the environment (Kempf et al. 2022; Slater et al. 2016), increasing the potential for onward transmission. It is typically assumed that shedding intensity is directly proportional to the intensity of infection (Beldomenico 2020; Chase‐Topping et al. 2008; DiRenzo et al. 2014; Kao et al. 2006), with hosts supporting the highest parasite burden having the highest shedding.

However, other factors might modulate the efficiency of shedding. Notably, co‐infection (i.e., being infected by multiple parasites at a given time) has been suggested to promote higher shedding intensities (Cattadori et al. 2014; Lass et al. 2013) and increased transmission (Susi et al. 2015). For example, in an experimental study of laboratory mice co‐infected with a bacterial pathogen (*Bordatella bronchiseptica*) and a helminth parasite (*Heligmosomoides polygyrus*), co‐infection promoted increased infection intensities, higher chronic shedding of helminth eggs, and the emergence of supershedders (see Table 1 for definition) (Lass et al. 2013). Similarly, in a natural population of European rabbits, co‐infection with two helminths (*Trichostrongylus retortaeformis* and *Graphidium strigosum*) altered the shedding dynamics of *G. strigosum*, resulting in higher shedding in co‐infected individuals (Cattadori et al. 2014).

Underlying co‐infections present a constant immune challenge to the host with vast immunological repercussions (Graham et al. 2007; King et al. 2006; Knowles 2011). Such immunological changes may affect how hosts and parasites interact, presenting a mechanism by which co‐infection may promote supershedding. Alternatively, or in addition, interactions among co‐infecting parasites may affect plastic life history decisions. It has been demonstrated that parasites alter their reproductive investment in response to environmental change (Greischar et al. 2016). Changes to the within‐host environment during co‐infection may increase perceived mortality risk to the parasite and trigger terminal investment in reproduction (Clutton‐Brock 1984), leading to higher shedding. Consistent with this hypothesis, it has been found that changes in traits related to parasite fecundity increase shedding intensity (Ghosh et al. 2018), and in co‐infected hosts, parasites demonstrate exaggeration of traits associated with reproduction (Ahmed et al. 2017; Cattadori et al. 2014).

In this study, we used a gastrointestinal helminth–gamebird system to quantify the relative importance of infection intensity vs. co‐infection in shaping shedding intensity. Ring‐necked pheasants ( *Phasianus colchicus* ) are released in large numbers to the UK countryside for game shooting (Robertson et al. 2017), with various ecological consequences (Sage et al. 2020). This study was part of a larger project that addresses the impact of pheasant releases on host–parasite interactions. *Heterakis gallinarum* is an intestinal helminth of gallinaceous birds and highly prevalent in ring‐necked pheasants (Cupo and Beckstead 2019; Draycott et al. 2000), with a faecal‐oral transmission route and a direct life cycle (Clapham 1933; Cupo and Beckstead 2019). Ring‐necked pheasants are also hosts to a range of other endoparasites in their digestive tract (for example hairworms (*Capillaria* spp.) (Draycott et al. 2000; Goldová et al. 2006) or *Eimeria* spp., the causative agent of coccidiosis (Clapham 1933; Goldová et al. 2006)) which can be used to investigate the influence of co‐infection. In this context, if shedding is primarily influenced by parasite burden, we predict a strong positive association between the two. Alternatively, if co‐infection alters parasite life history decisions through host–parasite and/or parasite–parasite interactions, we predict a disassociation between parasite burden and shedding intensity and increased shedding in co‐infected hosts.

## 2 Methods

### 2.1 Study Sites and Sample Collection

For this study, pheasants were collected postmortem between November and December 2023 from 8 recreational pheasant shoots across south‐west England (Figure S1). Pheasants were sexed and body size (beak—cloaca length, to the nearest 0.5 cm) and body mass (to the nearest 0.5 g) were recorded. Scaled mass index (SMI) was calculated as a measure of body condition following (Peig and Green 2009). We calculated SMI separately for males and females to account for sexual size dimorphism (Whiteside et al. 2018).

A faecal sample was collected directly from the cloaca and refrigerated at 4°C within 10 h of collection. Faecal samples were stored for a maximum of 5 days before processing. The lower digestive tract (small and large intestine, including the caeca, colon and cloaca) was removed from the same individuals and immediately stored in 10% formalin. In total, faecal samples and intestines from 58 pheasants were obtained (Table S1).

This research was conducted under the approval of the Ethical Committee of the University of Exeter (ethical approval number 513904). The study was conducted in accordance with all relevant ethical regulations and principles. All pheasants were released under licence and killed under the Game Act 1831.

### 2.2 Quantification of Shedding Intensity

A modified McMaster method was used to quantify shedding intensity (Levecke et al. 2011). 0.5 g of faecal matter was suspended in 7 mL of sodium nitrate flotation solution (specific gravity 1.2 +/− 0.05) (VetLab Supplies). The suspended sample was homogenised and strained to remove any large debris. 0.5 mL aliquots were added to two slide chambers on a McMaster slide. Slides were visually examined using light microscopy at 40× magnification. The number of *H. gallinarum* eggs on each slide was recorded. Slides were also examined for evidence of secondary helminth infections and the presence of *Eimeria* spp. oocytes. Published keys assisted in the morphological identification of helminth eggs and *Eimeria* spp. oocytes (Deviyanti et al. 2023; Goldová et al. 2006; Metwally et al. 2020). Eggs of *S. trachea* and *Capillaria* spp. are challenging to visually distinguish under 40× magnification. Unfortunately, respiratory tracts were not available for direct *S. trachea* quantification. Therefore, evidence of these infections was recorded as only the presence or absence of co‐infecting helminths. Counts were expressed as eggs per gram (EPG) and oocytes per gram (OPG), for helminths and *Eimeria* spp., respectively. This was obtained by multiplying the sum of both chambers by 50.

### 2.3 Quantification of Infection Intensity

Intensity of infection was quantified as the number of helminths present in the lower digestive tract. For each sample, the digestive tract was opened longitudinally and flushed with running water over a fine mesh sieve (aperture of 150 *mic*). Helminths collected in the sieve were retained, identified (Tanveer et al. 2015) and counted. The lining of the digestive tract was also examined. *H. gallinarum* and *Capillaria* spp. were observed in the sampled pheasants. Infection intensity quantification was conducted blind with respect to faecal egg counts.

### 2.4 Statistical Analyses

We used a generalised linear model with a negative binomial error structure to identify predictors of *H. gallinarum* shedding. Overdispersion was assessed by fitting an initial Poisson model and inspected using the DHARMa package (Hartig 2018). The Poisson model showed strong overdispersion (*dispersion parameter* = 193.05, *p* < 0.001). The final negative binomial model showed no significant overdispersion (*dispersion parameter* = 2.09, *p* = 0.072). *H. gallinarum* EPG of faeces was included as the response variable, and counts of *H. gallinarum* adults found in the digestive tract, host sex, host body condition, sampling location and co‐infection status (co‐infection/no co‐infection) were included as explanatory variables. The duration of faecal sample storage was included as an additional covariate to account for possible sample degradation over time (Crawley et al. 2016).

Second, we explored the role of co‐infection on *H. gallinarum* shedding in more detail using generalised linear models with a negative binomial error structure. All models included the same variables as the initial model but varied in their measure of co‐infection: (a) co‐infection with another helminth (yes/no), (b) co‐infection with *Eimeria* spp. (yes/no) and (c) the number of detected co‐infections (none, one, two).

Finally, we used a generalised linear model with a zero‐inflated negative binomial error structure to identify predictors of *H. gallinarum* infection intensity. This error structure was used to account for overdispersion, and high excess zero counts in the data (dispersion parameter = = 0.88998, *p* = 0.760). Counts of *H. gallinarum* in the digestive tract were included as the response variable and host sex, host body condition, sampling location and co‐infection status were included as explanatory variables.

All models were fitted using an ordinary least squares framework. All models were inspected for homogeneity of variance, normality of error structures, linearity and overdispersion via simulated model residuals using DHARMa (Hartig 2018). All models met their assumptions. Significance of factors was obtained by comparing two nested models, with or without variables of interest, using likelihood ratio tests. Host body condition and *H. gallinarum* infection intensity were scaled to aid model conversion.

All analyses were conducted in R version 4.3.0 (R Core Team 2024) using the packages lme4 (Bates et al. 2015), tidyverse (Wickham et al. 2019), DHARMa (Hartig 2018), ggplot2 (Wickham 2016), MASS (Venables and Ripley 2002), patchwork (Pedersen 2019), glmmTMB (Brooks et al. 2017) and gg.gap (Lou et al. 2019).

## 3 Results

### 3.1 Prevalence of Infections

All 58 individuals were infected with *H. gallinarum*. 11 individuals displayed evidence of *H. gallinarum* infection in faecal examinations only, and 1 individual displayed evidence of infection in only intestinal dissection. The average intensity of infection was 35 (8 ± SE) *H. gallinarum* adults per host. The average shedding intensity was 545 EPG (91 ± SE) (Table 2), however, this value varied significantly in relation to the co‐infection status of the host (Table 2). Co‐infection was detected in 30 individuals (52%) (Table 2). Of these co‐infected individuals, 8 were infected with a second helminth only (27%), 9 with *Eimeria* spp. only (30%), and 13 with both *Eimeria* spp. and a second helminth (43%) (Table 2).

**TABLE 2:** The average EPG of *H. gallinarum* in singularly and co‐infected hosts. ‘*N*' denotes the number of hosts in each group, ‘%’ denotes the percentage of the sample population this represents, ‘Range’ is the highest and lowest EPG count observed in each group, ‘SE is the standard error in EPG around the mean. The total sample size is 58.

Group	*N*	%	Mean	Range	SE ±
All individuals	57	98	545	0–5500	91
Co‐infected (any)	30	52	728	50–5200	165
Singularly infected	28	48	348	0–550	44
Co‐infected infected (helminth only)	8	14	544	50–1550	164
Co‐infected (protozoa only)	9	16	545	50–1150	93
Co‐infected (helminth and protozoa)	13	22	969	50–5200	360

### 3.2 Determinants of *H. gallinarum* Shedding

**FIGURE 1:**
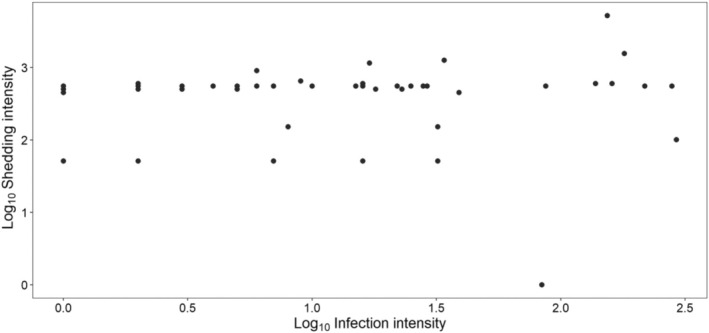
The relationship between *H. gallinarum* infection intensity and *H. gallinarum* shedding intensity. Shedding intensity is the EPG of *H. gallinarum* in faecal matter of ring‐necked pheasants. Infection intensity is the count of *H. gallinarum* adults in the gastrointestinal tract of ring‐necked pheasants.

**FIGURE 2:**
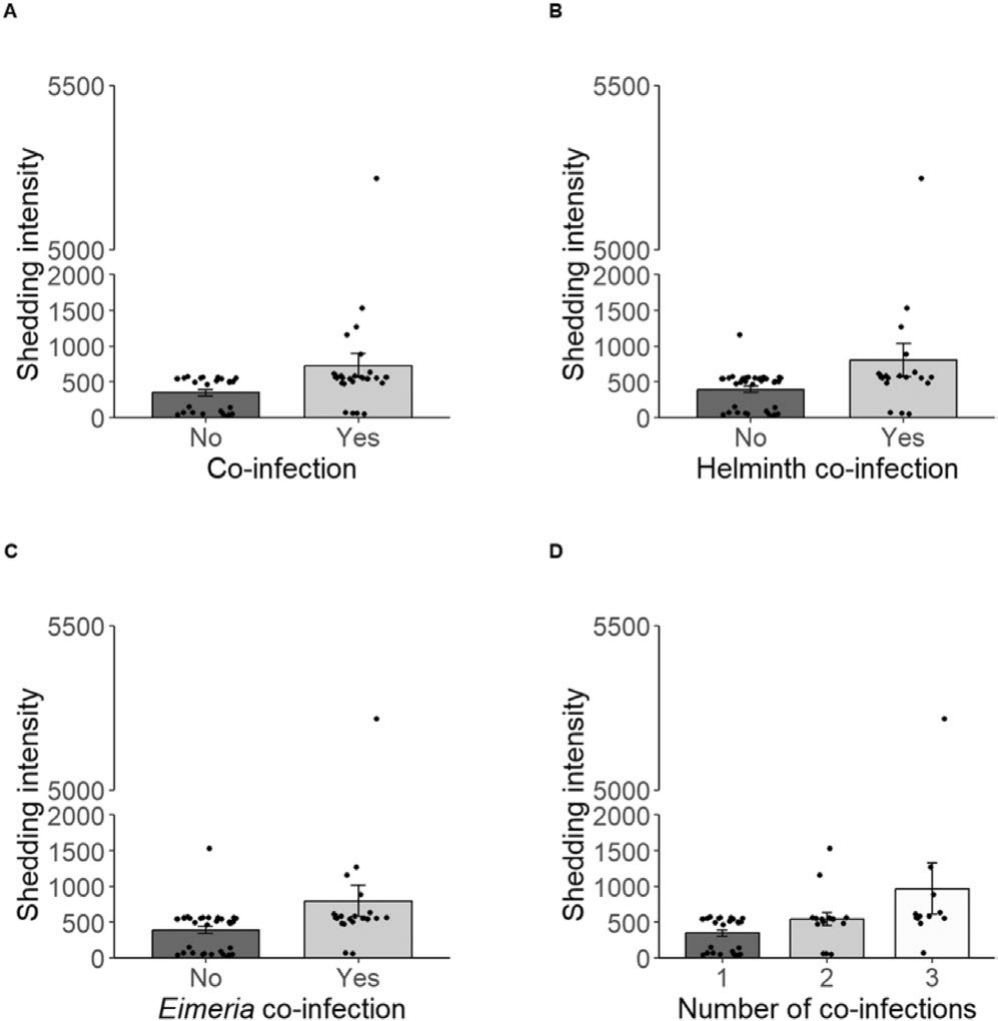
The effects of co‐infection on *H. gallinarum* shedding intensity (EPG) in ring‐necked pheasants. Panels A–C show the differences between mono‐infected and co‐infected hosts with (A) any other endoparasite, (B) a secondary helminth species, (C) *Eimeria* spp. (D) The cumulative effect of supporting multiple co‐infections. Bars represent the mean, error bars represent standard error and points represent individual data points.

We found no association between *H. gallinarum* infection intensity and *H. gallinarum* shedding intensity (*ꭓ* ^2^_1_ = 0.382, *p* = 0.537; Figure 1). Instead, shedding intensity was influenced by the co‐infection status of the host (*χ* ^2^_1_ = 6.012, *p* = 0.014; Figure 2a). We found that *H. gallinarum* shedding intensity was higher in hosts co‐infected with another helminth species (*χ* ^2^_1_ = 7.080, *p* = 0.008; Figure 2b). Similarly, hosts co‐infected with *Eimeria* spp. had increased *H. gallinarum* shedding (*χ* ^2^_1_ = 8.328, *p* = 0.004; Figure 2c). These co‐infection effects were cumulative, with the intensity of *H. gallinarum* shedding increasing with increasing number of co‐infections (*χ* ^2^_1_ = 14.065, *p* = 0.009; Figure 2d).

**FIGURE 3:**
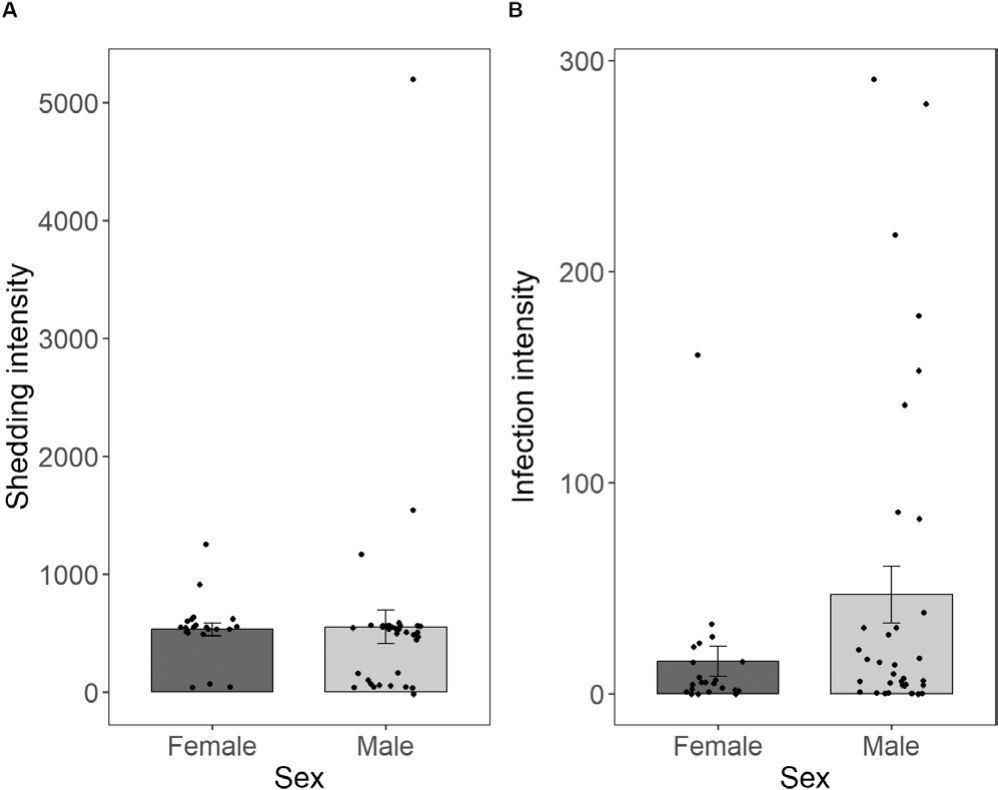
The effect of host sex on (A) *H. gallinarum* shedding intensity and (B) *H. gallinarum* infection intensity in ring‐necked pheasants. Shedding intensity is the EPG of *H. gallinarum* in faecal matter of ring‐necked pheasants. Infection intensity is displayed as the number of *H. gallinarum* present in the lower digestive tract of ring‐necked pheasants. Bars represent the mean, error bars represent the standard error and dots represent individual data points.

There were no differences in *H. gallinarum* shedding between male and female hosts (*ꭓ* ^2^_1_ = 0.398, *p* = 0.528; Figure 3a) or in relation to host body condition (*ꭓ* ^2^_1_ = 0.013, *p* = 0.912). A large proportion of variation in shedding was explained by sampling location (*ꭓ* ^2^_7_ = 49.505, *p* < 0.001) and *H. gallinarum* EPG decreased with increasing faecal sample storage duration (*ꭓ* ^2^_1_ = 6.302, *p* = 0.012; Figure S2).

### 3.3 Determinants of *H. gallinarum* Infection Intensity

Co‐infection status of the host had no effect on *H. gallinarum* infection intensity (*χ* ^2^_1_ = 0.704, *p* = 0.402), but infection intensity was higher in male hosts compared to female hosts (*χ* ^2^_1_ = 4.268, *p* = 0.039; Figure 3b). No association between host body condition and *H. gallinarum* infection intensity was found (*χ* ^2^_1_ = 0.665, *p* = 0.415). A large proportion of variation in infection intensity was explained by sampling location (*χ* ^2^_7_ = 23.921, *p* = 0.001).

## 4 Discussion

Superspreaders contribute disproportionately to the onward transmission of disease (Lloyd‐Smith et al. 2005), yet the drivers of transmission heterogeneity among hosts remain poorly understood. Our study shows that intensity of infection is a poor predictor of shedding intensity in a gastrointestinal helminth of gamebirds. Instead, we found that co‐infection with other endoparasites (*Capillaria* spp., *S. trachea* and/or *Eimeria* spp.) was the primary trait associated with increased *H. gallinarum* shedding. Overall, our results show that host shedding cannot be explained by infection intensity alone but is the result of complex relationships between hosts and their parasites.

Our results are in line with previous studies which documented a disassociation between parasite burden and shedding (Cattadori et al. 2014; Ghosh et al. 2018; Lass et al. 2013). Interestingly, a mismatch is most commonly documented in helminth systems (Cattadori et al. 2014, Ghosh et al. 2018, Lass et al. 2013), whereas the association seems to hold for bacteria (Lass et al. 2013; Pathak et al. 2010). The comparatively complex life histories of helminths (Benesh et al. 2014; De Paepe and Taddei 2006; Wang 2017), and long, sub‐clinical nature of infections (MacDonald Andrew et al. 2002; Maizels et al. 2004), may explain these differences.

Differential effects of host sex on infection intensity and shedding, respectively, further strengthened the conclusion that the two are not intrinsically linked. Whereas male pheasants had higher infection intensities than females, a pattern typically explained by physiological or behavioural differences between the sexes (Klein and Flanagan 2016; Poulin 1996; Robinson et al. 2008; Schuurs and Verheul 1990), this male bias in infection intensity did not translate into higher male shedding.

Modulation of the host immune system by co‐infecting pathogens may cause the disassociation between infection intensity and shedding. Parasites and pathogens are known to antagonise the host immune system (Fenton 2008; Fenton et al. 2008; King et al. 2006; Kutzer and Armitage 2016). For example, helminth infections induce the release of immunoregulatory monocytes and immunosuppressive cytokines (Allen and Maizels 2011), which downregulate immune function. Simultaneous impacts on host immune function by multiple co‐infecting parasites and pathogens might disproportionally lower host resistance, allowing individual parasites to increase their reproductive output, leading to higher shedding.

Alternatively, changes in parasite life history strategies in co‐infected hosts might cause the observed pattern. Terminal investment theory predicts that when an individual senses increased mortality risk, it should increase investment in reproduction (Clutton‐Brock 1984). Under this hypothesis, changes to the within‐host environment during co‐infections (e.g., increased competition for resources) may cause individuals to prioritise reproduction, leading to increased shedding. Previous studies have shown that in co‐infected hosts parasites are larger (Cattadori et al. 2014; Lass et al. 2013), and have more eggs in utero (Ahmed et al. 2017; Cattadori et al. 2014), supporting both of these scenarios.

This study is correlative and provides only a temporal snapshot of infection as we can only quantify host shedding at the time of death over a relatively short shooting season period. Given that infections are temporally stochastic (Cattadori et al. 2014) with high seasonal variation (Ara et al. 2021), a time‐series approach in living hosts and/or experimental manipulation of infection would be an exciting next step to disentangle these two scenarios further and identify the mechanisms by which co‐infection increases shedding rates.

This study did not differentiate between two nematode species, *Capillaria* spp. and *S. trachea*, so it may have underestimated the number of co‐infections in some hosts. It is also possible that hosts were infected with additional co‐infecting parasites that were not detected with the methods used in this study. Future work capturing a comprehensive overview of total parasite richness at a given time would provide further insight into the role of co‐infection in modulating shedding patterns.

To conclude, our results show that co‐infection is a key driver of variation in shedding intensity, whereas infection intensity is a poor predictor of shedding intensity. This highlights the key role of host–parasite and/or parasite–parasite interactions in shaping onward transmission of disease.

## Author Contributions

**Katherine Prescott:** conceptualization (equal), data curation (lead), formal analysis (lead), investigation (lead), methodology (lead), project administration (equal), resources (supporting), visualization (lead), writing – original draft (lead), writing – review and editing (supporting). **Emile Michels:** conceptualization (equal), data curation (supporting), investigation (supporting), methodology (supporting), project administration (equal), resources (lead), supervision (supporting), writing – review and editing (supporting). **Barbara Tschirren:** conceptualization (equal), formal analysis (supporting), funding acquisition (lead), supervision (lead), validation (lead), writing – review and editing (lead).

## Disclosure

Statement on inclusion: Our study was conducted in the Southwest of England. Our research team includes scientists from the region. We will share outcomes from the research with gamekeepers and shoot owners involved in the project.

## Conflicts of Interest

The authors declare no conflicts of interest.

## Supporting information


Appendix S1.

## Data Availability

All data and code are available in Dryad (https://doi.org/10.5061/dryad.sqv9s4ndd).
