# Supplementary material for: Co‐Infection, but Not Infection Intensity, Increases Shedding in a Gastrointestinal Helminth of Gamebirds
Source: Ecol Evol. 2025 Jun 5;15(6):e71483. doi: 10.1002/ece3.71483 (PMC12141756; doi:10.1002/ece3.71483)
Supplement: Supplementary file 1 — Appendix S1. [file ECE3-15-e71483-s001.docx]

Supplementary materials for

**Co-infection, but not infection intensity, increases shedding in a gastrointestinal helminth of gamebirds**


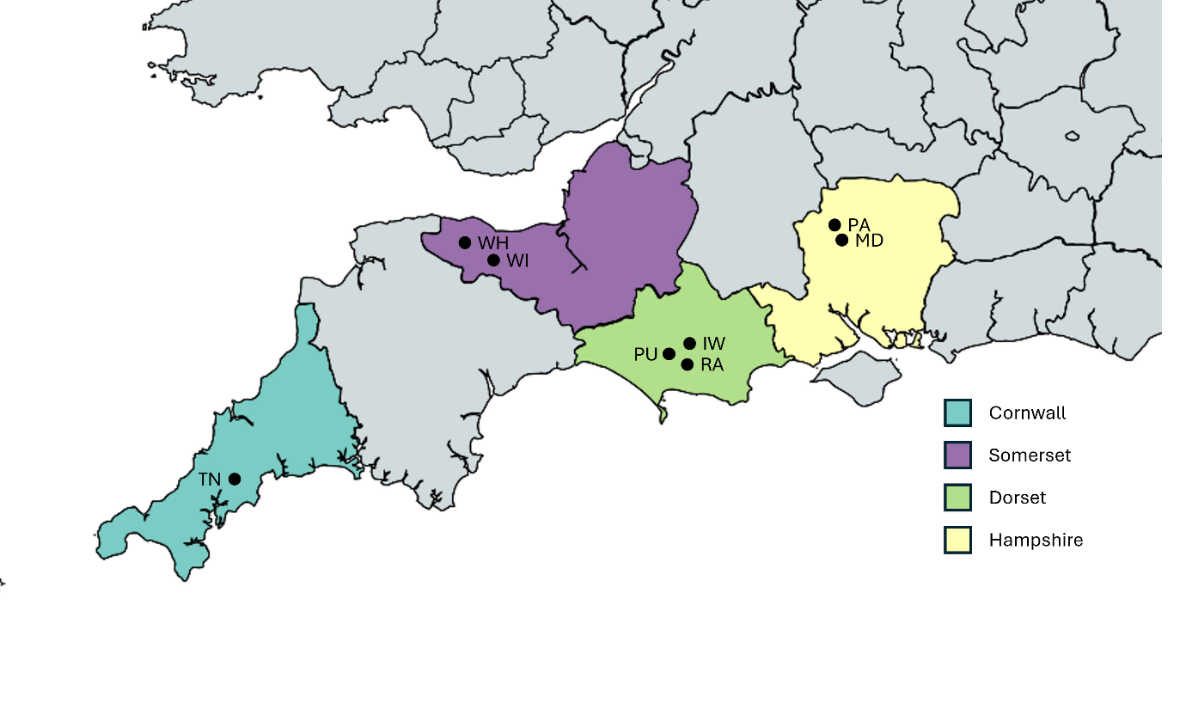


**Figure S1.** Map displaying the approximate locations of ring-necked pheasant collection sites. Samples were collected from 1 site in Cornwall (TN), 2 sites in Somerset (WH, WI), 3 sites in Dorset (PU, IW, RA), and 2 sites in Hampshire (PA, MD).

**Table S1.** The number of ring-necked pheasants collected from each site. Table shows the number of viable samples collected. Viable samples were those with a minimum of 0.5g faecal material. Site codes are consistent with those used in Figure S1.

| **Site** | ***Males*** | ***Females*** | ***Total*** |
| --- | --- | --- | --- |
| IW | 6 | 2 | 8 |
| MD | 5 | 3 | 8 |
| PA | 0 | 3 | 3 |
| PU | 0 | 3 | 3 |
| RA | 8 | 1 | 9 |
| TN | 2 | 2 | 4 |
| WH | 7 | 5 | 12 |
| WI | 8 | 3 | 11 |
| **Total** | **36** | **22** | **58** |

**
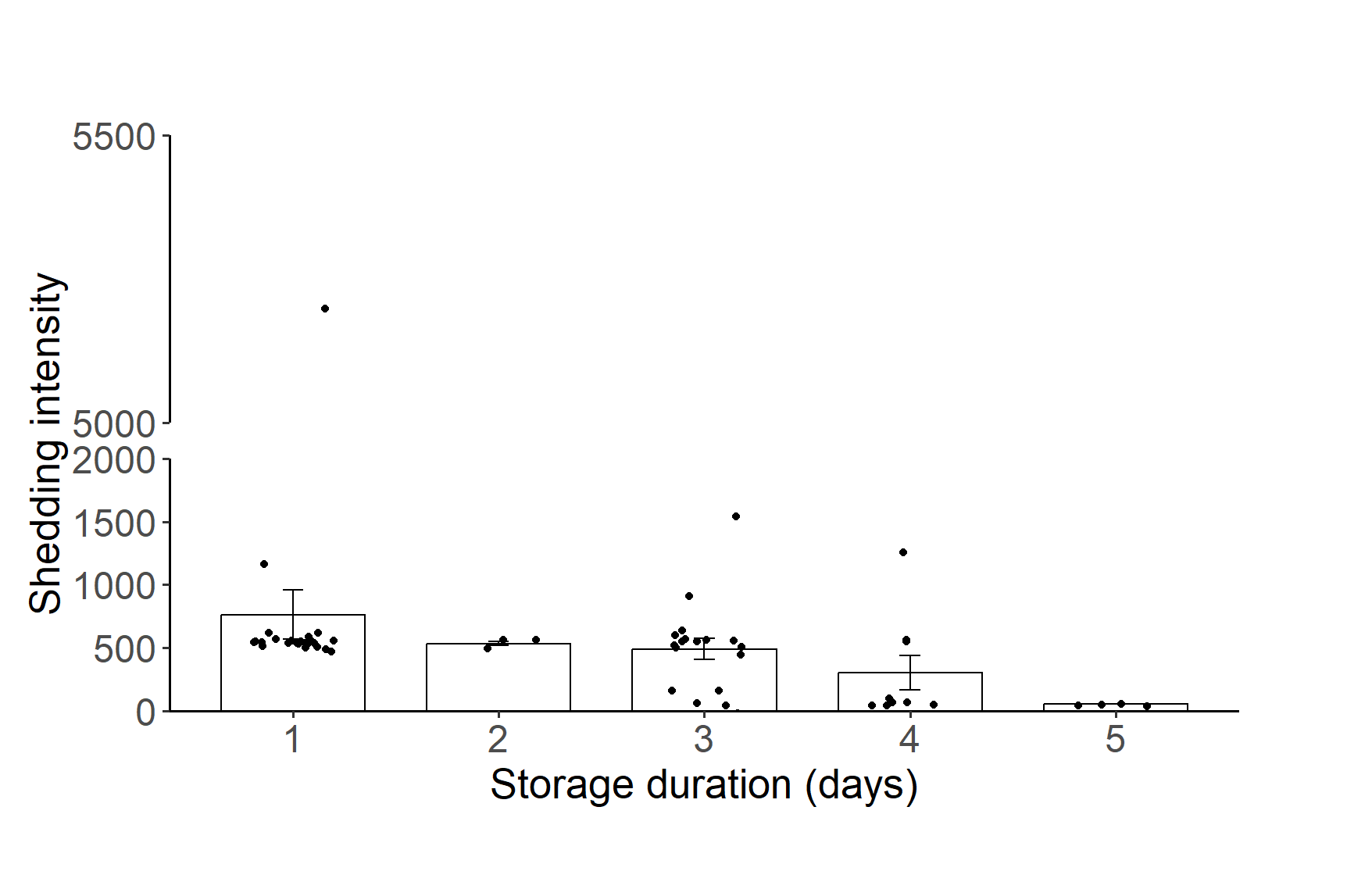
**

**Figure S2.** *H. gallinarum* shedding intensity in relation to sample storage duration. Shedding intensity is displayed as the EPG of *H. gallinarum* in faecal samples of ring-necked pheasants. Storage duration denotes the duration of faecal storage in 4°C refrigerated conditions in days. The bar represents the mean, error bars represent standard error, dots represent individual data points.
